# Inference of a causal relation between low-density lipoprotein cholesterol and hypertension using mendelian randomization analysis

**DOI:** 10.1186/s40885-021-00162-6

**Published:** 2021-02-26

**Authors:** Tae-Hwa Go, Kyeong Im Kwak, Ji-Yun Jang, Minheui Yu, Hye Sim Kim, Jang Young Kim, Sang Baek Koh, Dae Ryong Kang

**Affiliations:** 1 Center of Biomedical Data Science, Yonsei University Wonju College of Medicine, Wonju, Republic of Korea; 2 Department of Biostatistics, Yonsei University Wonju College of Medicine, Wonju, Republic of Korea; 3 Center of Cancer Data, National Cancer Center, Ilsan, Republic of Korea; 4 SENTINEL Team, Division of Endocrinology, Department of Internal Medicine, Yonsei University College of Medicine, Seoul, Republic of Korea; 5 Artificial Intelligence BigData Medical Center, Yonsei University Wonju College of Medicine, Wonju, Republic of Korea; 6 Department of Cardiology, Yonsei University Wonju College of Medicine, Wonju, Republic of Korea; 7 Institute of Genomic Cohort, University Wonju College of Medicine, Wonju, Republic of Korea; 8 Department of Preventive Medicine, Yonsei University Wonju College of Medicine, Wonju, Republic of Korea; 9 Department of Precision Medicine, Yonsei University Wonju College of Medicine, 20 Ilsan-ro, Wonju-si, Gangwon-do 26426 Wonju, Republic of Korea

**Keywords:** Low-density lipoprotein cholesterol, Hypertension, Mendelian randomization, Genetic epidemiology

## Abstract

**Background:**

It is known in some studies that higher the LDL-C, the greater the risk of developing cardiovascular disease. However, studies of the causal effects between LDL-C and hypertension are limited by their observational study design, and genetic epidemiology studies of associations between LDL-C and hypertension are lacking, as are studies using data for Koreans. In this study, we confirmed the causal effect of LDL-C on hypertension using Korean chip data.

**Method:**

The epidemiology and genotype data were collected from the Korean Genome and Epidemiology Study conducted by the Korea National Institute of Health and covered 20,701 subjects. Single-nucleotide polymorphisms associated with LDL-C were selected (*p-value* < 5 × 10^− 8^) from the Global Lipids Genetics Consortium database, and Mendelian randomization analysis (MRA) was performed with counted genetic risk scores and weighted genetic risk scores (WGRSs) for 24 single-nucleotide polymorphisms.

**Result:**

The assumptions for MRA were statistically confirmed, and WGRSs showed a strong association with LDL-C. Interestingly, while the relationship between LDL-C and hypertension was not statistically significant in the observational study, MRA study demonstrated that the risk of hypertension increased as LDL-C increased in both men and women.

**Conclusions:**

The results of this study confirmed that the relationship between LDL-C and hypertension is greatly influenced by genetic information.

**Supplementary Information:**

The online version contains supplementary material available at 10.1186/s40885-021-00162-6.

## Background

Hypertension, the most common cardiovascular disease in older adults, is one of the most important risk factors for cardiovascular diseases, including myocardial infarction, stroke, congestive heart failure, terminal renal disease, and peripheral vascular disease [1]. According to the World Health Organization, about 17 million people worldwide die from cardiovascular diseases, and about 9.4 million people die from hypertension. The prevalence of hypertension is expected to increase from 26% in 2000 to 29.2% in 2025, about 29% worldwide. About half of all older adults in Korea are estimated to have hypertension [2, 3], increasing medical expenses in older adults and negatively affecting the quality of life of both the patient and their family [4, 5]. As prevention of hypertension can alleviate the overall disease burden on society and improve quality of life, further research into hypertension prevention is needed, and since hypertension, a chronic disease, affects a number of factors, causal inference study of disease occurrence should incorporate genetic factors in addition to environmental factors [6].

In accordance with Mendel’s second law, genetic factors can indirectly affect disease incidence through various risk factors, making it necessary to identify causal associations through Mendelian randomization analysis (MRA). Mendelian randomization reflects the natural, random assortment of genetic variants during meiosis, yielding a random distribution of genetic variants in a population [7], and has been used in epidemiologic studies to identify causal relationships between risk factors and outcomes when causal confounding or reverse causality may interfere with causality inference [7–10]. To determine the genetic basis of a phenotype or to characterize gene function, conventional studies in genetic epidemiology seek to document associations between genetic and phenotype variations within a population. In such studies, genetic variations are assessed using markers, often single nucleotide polymorphisms (SNPs), and markers are considered informative if they show sufficient variation within a population and are of high enough prevalence to allow for meaningful comparisons. Meanwhile, it is also possible to exploit the random assignment of genes as a means of reducing confounding when examining exposure–disease associations: this is Mendelian randomization in the epidemiological context [7].

It is known in some studies that higher the LDL-C, the greater the risk of developing cardiovascular disease. Also, intervention trials using statins to lower LDL cholesterol have consistently reported substantial reductions in major cardiovascular events in treated groups. However, these results were gleaned from epidemiological studies that did not include genetic factors [11–17]. Moreover, very few studies have been conducted in Asians.

Thus, in this study, we performed Mendelian randomization using Korean chip data to investigate the existence of causal effects between LDL-C and hypertension.

## Method

### Study population

**Fig. 1:**
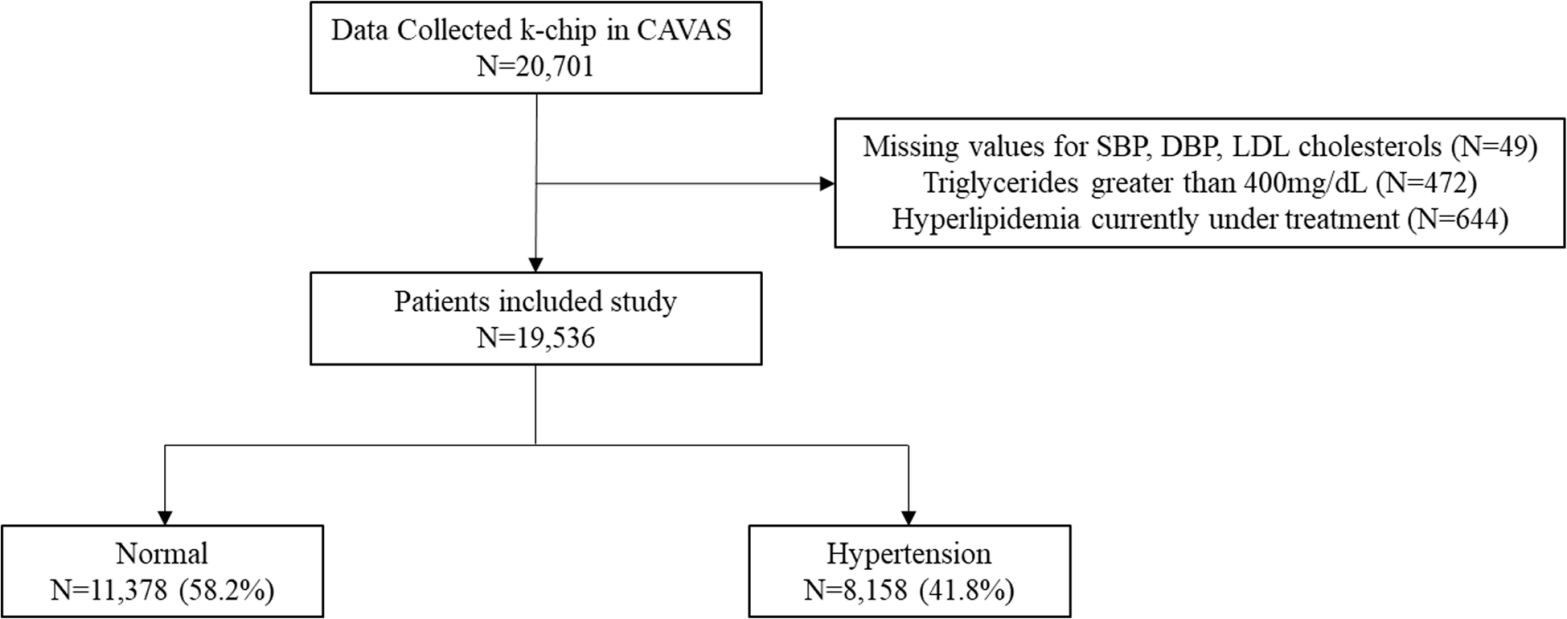
Flow chart of the study. CAVAS, Cardiovascular Disease Association Study, *SBP* systolic blood pressure, *DBP* diastolic blood pressure, *LDL-C* low-density lipoprotein cholesterol

This study evaluated participants included in a rural-based, cardiovascular disease association study (CAVAS) among individuals of the Korean Genome Epidemiology Study (KoGES) conducted by the Korea Centers for Disease Control and Prevention. The CAVAS study covered the years 2005–2011 and recruited men and women aged 40–69 years living in 11 rural areas. A total of 28,338 people were recruited. Among them, 20,701 were surveyed for both epidemiological and genomic data. In this study, individuals who lacked information on systolic blood pressure (SBP), diastolic blood pressure (DBP), or LDL-C (*n* = 49) and those with triglycerides levels greater than 400 mg/dL were excluded (*n* = 472) [18]. Except for 644 subjects currently undergoing treatment for hyperlipidemia, a total of 19,536 subjects were analyzed in this study (Fig. 1). The study protocol was approved by the Institutional Review Board of Wonju Severance Christian Hospital (CR317334).

### Data collection

Study participants were asked to complete self-reported questionnaires in order to assess their personal and family medical histories, smoking habits, alcohol consumption, exercise status, and use of medication. Smoking status and drinking status were categorized as never, past, or current. Height, body weight, and waist circumference were measured using standard methods. Waist circumference was measured at the narrowest point between the upper iliac crest and the lowest rib after normal expiration. Blood pressure was measured by averaging three recordings taken in the morning after at least 10 min of rest in a sitting position. Laboratory samples were obtained after a 12-h fast. Plasma total cholesterol, triglycerides, high-density lipoprotein cholesterol (HDL-C), creatinine, and alanine and aspartate aminotransferase levels were measured using a Hitachi 747 chemistry analyzer (Hitachi Ltd., Tokyo, Japan). LDL-C was assessed using the Friedewald equation. Nutrition was examined using data extracted from the Korea Health and Nutrition Examination Survey on multi-frequency foods in 1988 considering the contributions of each of the 17 major nutrients.

### Gene data source

Genetic data were gleaned from next-generation sequencing and SNP information contained in K-CHIP (Center for Genetic Studies, Genome Center, Korea National Institute for Disease Control and Prevention). The K-CHIP comprises 830,000 representative SNPs in the Korean genome extracted from next-generation sequencing of more than 2000 Asian genomes and 1000 Korean genomes. Currently, K-CHIP covers about 95% of SNPs, with a genome representation of 5% or more [19].

### Hypertension and LDL-C

Hypertension was defined in accordance with the Korean Society of Hypertension 2018 treatment guidelines [20]: 1) SBP ≥ 140 mmHg, 2) DBP ≥ 90 mmHg, or 3) currently undergoing treatment for hypertension. LDL-C was categorized as optimal (< 100 mmHg), near optimal (100–219 mmHg), borderline high (130–159 mmHg), high (160–189 mmHg), and very high (≥ 190 mmHg) as indicated by the National Cholesterol Education Program Adult Treatment Panel III. In the present study, LDL-C was analyzed as optimal (< 100 mmHg), near optimal (100–219 mmHg), and high (≥ 130 mmHg).

### Gene selection (genotype)

Genes related to LDL-C was selected with reference to the Global Lipids Genetics Consortium (GLGC). Based on genome-wide association study results, we selected genes with *p-value*s < 5 × 10^− 8^ for association between SNPs and LDL-C and with low linkage disequilibrium. Of these, haplotypes were excluded. In total, 24 SNPs were selected for analysis.

### Statistical analysis

To analyze differences in the general characteristics of the study subjects according to the presence of hypertension, t-test was used for continuous variables, and chi-square test was used for categorical variables.

Three analytical methods were used to confirm the relationship between LDL cholesterol and hypertension. In the first method, logistic regression analysis was performed to confirm relationships noted in observational study analysis. The second and third methods implemented Mendelian randomization for two-stage least square regression using counted genetic risk scores and weighted genetic risk scores, respectively. In total, three models were developed: model 1 was unadjusted; model 2 was adjusted for age, family history of hypertension, and body mass index; and model 3 was adjusted for the same covariates in model 2 in addition to smoking status, drinking status, and salt intake.

Before implementing MRA, three basic assumptions were proposed: Assumption 1 assumed that the instrumental variable would be associated with the exposure of interest. Assumption 2 assumed that the instrumental variable is dependent on factors confounding the association between exposure and the outcome. Assumption 3 assumed that the instrumental variable is only associated with the outcome through the exposure.

Assumption 1 was confirmed through F-statistics and indicated that SNPs identified by consortium were associated with LDL-C. Only SNPs with *p-value*s < 5 × 10^− 8^ were considered for analysis and confirmed LDL-C according to genotype through Cuzick’s test. In addition, genetic risk scores (GRSs) were calculated for the SNPs satisfying the assumption, and linear relationships for counted GRSs and weighted GRSs with LDL-C were confirmed. Assumption 2 indirectly confirmed that the two relationships were independent by identifying differences from confounding factors according to genotypes of each SNP because direct proof was impossible. Finally, assumption 3 was confirmed using the Durbin-Wu-Hausman test and Sargan test.

All analyses were conducted using SAS version 9.4 (SAS Institute Inc., Cary, NC, USA), R version 3.3.1. and STATA. *p-value*s < 0.05 were considered indicative of statistical significance.

## Results

### Baseline characteristics

For the 19,536 subjects included in this study (men: 7253; women: 12,283), hypertension was recorded in 8158 (41.8%). Compared with normal individuals, those with hypertension were older and had higher weight and waist circumference values. The mean ± SD values of LDL-C were 124.5 ± 31.5 and 125.8 ± 33.7 mg/dL in normal individuals and those with hypertension, respectively (Table 1).

**Table 1:** Baseline characteristics according to hypertension

Variables	Normal (*N* = 11,378)	Hypertension (*N* = 8158)	*p-value*
Sex			<.0001
Men	4077 (35.8)	3176 (38.9)	
Women	7301 (61.2)	4982 (61.1)	
Age	57.2 ± 9.7	60.9 ± 9.0	<.0001
Family history of hypertension	1937 (17.2)	2104 (26.1)	<.0001
Smoking status			<.0001
Never smoker	6367 (71.0)	4667 (70.9)	
Ex-smoker	1219 (13.6)	1059 (16.1)	
Current smoker	1386 (15.4)	853 (13.0)	
Alcohol drinking			0.0002
Never drinking	6069 (53.5)	4113 (50.5)	
Ex-drinking	782 (6.9)	628 (7.7)	
Current drinking	4500 (39.6)	3400 (41.8)	
Exercise			<.0001
< 3 times per weeks	878 (23.3)	514 (18.8)	
≥ 3 times per weeks	2888 (76.7)	2214 (81.2)	
Systolic blood pressure (mmHg)	116.2 ± 11.7	138.3 ± 17.0	<.0001
Diastolic blood pressure (mmHg)	73.9 ± 8.0	85.8 ± 11.0	<.0001
Body mass index (kg/m^2^)	23.9 ± 3.0	25.1 ± 3.2	<.0001
Waist circumference (cm)	82.5 ± 8.6	85.9 ± 8.7	<.0001
HDL-C (mg/dL)	45.7 ± 11.1	45.0 ± 11.1	<.0001
LDL-C (mg/dL)	124.5 ± 31.5	125.8 ± 33.7	0.0072
Total cholesterol (mg/dL)	195.8 ± 35.1	200.6 ± 36.8	<.0001
Triglyceride (mg/dL)	128.0 ± 64.9	148.9 ± 72.4	<.0001
Salt intake (mg)	2530.4 ± 1544.6	2470.1 ± 1517.2	<.0001

Values are presented as a number (percentage) or mean ± standard deviation

*SBP* Systolic blood pressure, *DBP* Diastolic blood pressure, *BMI* Body mass index, *HDL-C* High-density lipoprotein cholesterol, *LDL-C* Low-density lipoprotein cholesterol

### Association between genetic risk and LDL-C

The 24 genes chosen through the GLGC are listed in Table 2. *p-value* < 5 × 10^− 8^ was used to confirm the statistical significance of the relationship between individual genes and LDL-C (Assumption 1). F-statistics values for the relationship between genetic risk and LDL-C in relation to counted and weighted GRSs were 262.9 and 661.5, respectively, which is much higher than the standard F-statistics of 10. In both men (counted GRS: 74.7; weighted GRS: 161.6) and women (counted GRS: 152.4; weighted GRS: 384.0), the relationship between the genes and LDL-C was strong (Table 3). In addition, the association between gene polymorphism and LDL-C was examined (Supplement Table 1).

**Table 2:** List of 24 SNPs included to calculate genetic risk score for LDL-C

Chromosome	SNP	Gene	Risk allele	Other allele	β	SE(β)	F-statistic
1	rs41279716	CELSR2	A	T	0.0518	0.0065	20.27
1	rs4970834	CELSR2	C	T	0.1503	0.0047	33.26
1	rs79868705	CELSR2	G	A	0.0851	0.0126	10.47
1	rs79482788	CELSR2	G	A	0.0864	0.0126	10.49
1	rs12740374	CELSR2	G	T	0.1610	0.0044	44.21
1	rs35358959	PSRC1	G	A	0.0986	0.0088	18.94
1	rs672569	PSRC1	G	A	0.1431	0.0082	19.47
1	rs11596737	PDLIM1	G	A	0.0968	0.0088	20.86
1	rs17645031	MYBPHL	C	T	0.1004	0.0067	17.49
1	rs41306199	MYBPHL	C	T	0.0903	0.0091	17.49
11	rs651821	APOA5	C	T	0.0722	0.0094	10.94
11	rs7952602	ST3GAL4	C	G	0.0496	0.0054	10.67
16	rs8062041	TXNL4B	T	C	0.0250	0.0038	17.01
19	rs2738452	LDLR	G	A	0.0624	0.0053	19.06
19	rs2738464	LDLR	C	G	0.0422	0.0061	73.71
19	rs892114	SPC24	A	G	0.0353	0.0047	14.01
19	rs6511727	DOCK6	T	G	0.0266	0.0038	11.56
19	rs387976	NECTIN2	A	C	0.0818	0.0057	33.17
19	rs3852861	NECTIN2	G	T	0.0347	0.0041	17.18
19	rs7254892	NECTIN2	G	A	0.4853	0.0119	241.13
19	rs7412	APOE	C	T	0.5898	0.0101	307.79
19	rs445925	APOC1	G	A	0.3634	0.0081	206.40
19	rs56131196	APOC1	A	G	0.2011	0.0076	27.28
19	rs7259004	APOC1P1	G	C	0.2094	0.0092	30.97

*LDL-C* Low-density lipoprotein cholesterol, *SNP* Single-nucleotide polymorphism, *SE* Standard error

**Table 3:** F-statistic and *p-value*s for counted and weighted genetic risk scores

Genetic risk score	Counted GRS			Weighted GRS		
	F-statistic	R-square	*p-value*	F-statistic	R-square	*p-value*
All patients	262.9	0.013	< 2.2e-16	661.5	0.033	< 2.2e-16
Men	74.7	0.129	< 2.2e-16	161.6	0.028	< 2.2e-16
Women	152.4	0.016	< 2.2e-16	384.0	0.039	< 2.2e-16

*GRS* Genetic risk score

**Fig. 2:**
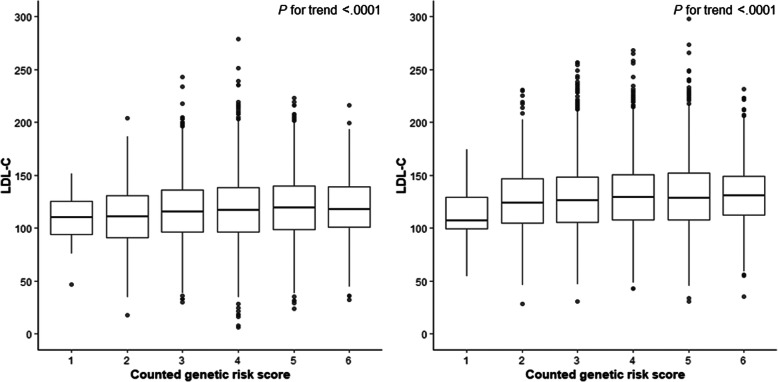
Association between LDL-C and counted genetic risk scores in men and women. *LDL-C* low-density lipoprotein cholesterol

Next, we examined differences in risk factors of hypertension according to *APOE* polymorphism (rs7412), which has the highest beta value in the GLGC (Assumption 2). In doing so, we noted statistically significant differences in LDL-C, HDL-C, total cholesterol, and triglyceride with *APOE* polymorphism (rs7412) (Table 4). In addition, we confirmed a trend of increasing LDL-C with increasing counted GRS (Fig. 2).

**Table 4:** Association between APOE (rs7412) genotype and potential confounders

Variables	Wild type (*N* = 17,153)	Heterozygous (*N* = 2316)	Homozygous (*N* = 67)	*p-value*
Sex				
Men	6373 (37.2)	853 (86.8)	27 (40.3)	0.8265
Women	10,780 (62.8)	1463 (63.2)	40 (59.7)	
Age	58.8 ± 9.6	58.6 ± 9.4	57.4 ± 10.3	0.4425
Family history of hypertension	3557 (20.9)	473 (20.6)	11 (16.4)	0.6169
Smoking status				0.7190
Never smoker	9695 (70.9)	1300 (71.6)	39 (66.1)	
Ex-smoker	2011 (14.7)	259 (14.3)	8 (13.6)	
Current smoker	1970 (14.4)	257 (14.2)	12 (20.3)	
Alcohol Drinking				0.8591
Never drinking	8923 (52.1)	1224 (52.9)	35 (52.2)	
Ex-drinking	1247 (7.3)	157 (6.8)	6 (9.0)	
Current drinking	6943 (40.6)	931 (40.3)	26 (38.8)	
Exercise				0.7963
< 3 times per weeks	1231 (21.6)	156 (20.6)	5 (19.2)	
≥ 3 times per weeks	4479 (78.4)	602 (79.4)	21 (80.8)	
Systolic blood pressure (mmHg)	125.6 ± 17.9	124.7 ± 17.5	124.2 ± 17.8	0.0912
Diastolic blood pressure (mmHg)	78.9 ± 11.1	78.6 ± 10.9	78.7 ± 10.1	0.5107
Body mass index (kg/m^2^)	24.4 ± 3.2	24.4 ± 3.2	24.0 ± 2.8	0.6311
Waist circumference (cm)	83.9 ± 8.8	84.0 ± 8.9	82.8 ± 8.7	0.5142
HDL-C (mg/dL)	45.2 ± 10.9	46.4 ± 12.2	46.3 ± 12.2	<.0001
LDL-C (mg/dL)	127.2 ± 32.3	110.2 ± 28.9	99.2 ± 44.8	<.0001
Total cholesterol (mg/dL)	199.6 ± 35.7	184.9 ± 33.2	176.4 ± 56.0	<.0001
Triglyceride (mg/dL)	136.1 ± 68.4	141.4 ± 72.0	154.8 ± 82.1	0.0002
Salt intake (mg)	2507.4 ± 1541.7	2499.0 ± 1482.4	2167.4 ± 1075.6	0.1897

Values are presented as a number (percentage) or mean ± standard deviation

*SBP* Systolic blood pressure, *DBP* Diastolic blood pressure, *BMI* Body mass index, *HDL-C* High-density lipoprotein cholesterol, *LDL-C* Low-density lipoprotein cholesterol

### Observational and Mendelian randomization analysis

In observational analysis, the risk of hypertension according to LDL-C was not statistically significant in Model 3, which was adjusted for confounding variables in men and women. In MRA using counted GRS, the risk of hypertension was statistically significant as LDL-C increased in all models. In Model 3, compared to optimal LDL-C, the odds ratios of hypertension risk were 1.07 (95% CI, 0.90–1.27) and 1.41 (95% CI, 1.08–1.84) for near optimal and high LDL-C levels, respectively, in men and 1.18 (95% CI, 1.03–1.35) and 1.83 (95% CI, 1.50–2.23) in women. Similar results were obtained in MRA using weighted GRS in model 3, with odds ratios for hypertension risk of 1.08 (95% CI, 0.91–1.27) and 1.42 (95% CI, 1.09–1.85) for near optimal and high group LDL-C levels, respectively, in men and 1.18 (95% CI, 1.03–1.35) and 1.84 (95% CI. 1.51–2.24) in women (Table 5).

**Table 5:** Association between LDL-C and hypertension in observational analysis and Mendelian randomization analysis

	Observational analysis	Mendelian randomization Analysis	
		Counted GRS	Weighted GRS
	OR (95% CI)	OR (95% CI)	OR (95% CI)
**Men**			
Model 1			
Optimal	1.00 (reference)	1.00 (reference)	1.00 (reference)
Near optimal	0.84 (0.75–0.95)	1.15 (0.97–1.37)	1.16 (0.97–1.37)
High	0.90 (0.79–1.01)	1.64 (1.25–2.16)	1.65 (1.26–2.17)
Model 2			
Optimal	1.00 (reference)	1.00 (reference)	1.00 (reference)
Near optimal	0.91 (0.79–1.04)	1.07 (0.90–1.26)	1.07 (0.90–1.27)
High	1.02 (0.89–1.17)	1.40 (1.07–1.83)	1.41 (1.08–1.84)
Model 3			
Optimal	1.00 (reference)	1.00 (reference)	1.00 (reference)
Near optimal	0.92 (0.80–1.05)	1.07 (0.90–1.27)	1.08 (0.91–1.27)
High	1.03 (0.90–1.18)	1.41 (1.08–1.84)	1.42 (1.09–1.85)
**Women**			
Model 1			
Optimal	1.00 (reference)	1.00 (reference)	1.00 (reference)
Near optimal	0.89 (0.79–0.99)	1.18 (1.03–1.35)	1.18 (1.03–1.36)
High	0.89 (0.80–0.99)	1.83 (1.51–2.23)	1.83 (1.50–2.23)
Model 2			
Optimal	1.00 (reference)	1.00 (reference)	1.00 (reference)
Near optimal	0.90 (0.79–1.02)	1.18 (1.03–1.35)	1.18 (1.03–1.36)
High	0.92 (0.81–1.04)	1.83 (1.51–2.23)	1.83 (1.50–2.23)
Model 3			
Optimal	1.00 (reference)	1.00 (reference)	1.00 (reference)
Near optimal	0.90 (0.79–1.02)	1.18 (1.03–1.35)	1.18 (1.03–1.35)
High	0.92 (0.81–1.03)	1.83 (1.50–2.23)	1.84 (1.51–2.24)

Model 1: adjusted for age and body mass index

Model 2: Model 1 + smoking and drinking status

Model 3: Model 2 + salt intake

*LDL-C* Low-density lipoprotein cholesterol, *GRS* Genetic risk score, *OR* Odds ratio

## Discussion

In this study, CAVAS was used to recruit men and women aged 40–69 years. Of the 19,536 patients analyzed in this study, 11,378 were normal, and 8158 had hypertension. The aim of this study was to demonstrate the causal relationship between LDL-C and hypertension using genetic analysis, and the relationship was confirmed by observational and MRA methods. In the observational study, the relationship between LDL-C and hypertension was not statistically significant; however, MRA showed that the risk of hypertension increased as LDL-C increased in both men and women. The main results of this study confirmed that the relationship between LDL-C and hypertension is influenced by genetic information.

The results from the observational studies and MRA were different in this study. This difference may have been caused by residual confounders not included in the observed regression model [21]. Since there may be an inverse relationship between elevated LDL-C in patients with hypertension, a causal relationship was inferred by reducing bias using genetic data.

Cholesterol is generally known as a risk factor for hypertension. However, most studies have only identified a relationship between HDL-C and cardiovascular risk; few have found LDL-C to affect incident hypertension. Otsuka et al. [22] reported the development of hypertension according to LDL-C quintiles. Therein, the risk of hypertension was 1.27 times higher at the highest quintile than the lowest. With the exception of one study conducted in China, the research by Otsuka et al. is the first to demonstrate a longitudinal association between lipid measures and the risk of incident hypertension in Asian individuals, and suggested that dyslipidemia is associated with an increased risk of incident hypertension. As mentioned in their article, the first mechanism potentially explaining the relationship between dyslipidemia and hypertension risk suggests that dyslipidemia impairs endothelial function, which can interfere with nitric oxide production and the control of blood pressure. Second, dyslipidemia can cause development of hypertension by decreasing baroreflex sensitivity. Third, dyslipidemia reduces the distensibility of large elastic arteries, and finally, a lack of physical activity or high-fat diet promotes obesity. In obese individuals, adipose tissue excessively secretes adipocytokines, resulting in insulin resistance and subsequent activation of the sympathetic nervous system and the renin-angiotensin system. These biological changes have been reported to lead to an increase in blood pressure. Our results support this mechanism and hold significance in confirming the causality between LDL-C and hypertension.

However, our study entails some limitations. First, LDL-C was not investigated in CAVAS; therefore, we used the Friedewald formula to calculate LDL-C. Notwithstanding, the National Health Screening Program of Korea also estimates LDL-C using the formula. Second, among the risk factors that could affect hypertension, there were a few that our study could not take into account. Third, generalizing the results of this study to all Koreans would be difficult. However, since the study was conducted using SNPs validated in the literature, we expect that any bias would be minimal. Finally, there is the potential for linkage disequilibrium and pleiotropy as a limitation of MRA [23–25]. Despite these limitations, genetic analysis based on MRA provides a way to overcome the possibility of interpreting causal conclusions in observational studies [26].

In summary, identifying causal relationships in observational studies is not easy. However, Mendelian randomization creates an environment through which causal associations can be identified without performing randomized controlled trials, which are expensive and time-consuming. Using MRA, we found that the relationship between LDL-C and hypertension is indeed causal, and further validation is needed using further next-generation sequencing analysis.

## Conclusions

In this study, a causal association between LDL-C and hypertension was confirmed using MRA. The causal effects of LDL-C and hypertension were confirmed using genetic information. Our results showed that the relationship between LDL-C and hypertension, which was stronger in women, is reflected in genetic risk scores.

## Supplementary Information

**Additional file 1: Supplement Table 1**. Associations between genetic polymorphism and LDL-C.

## Data Availability

The datasets used and/or analyzed during the current study are available from the corresponding author on reasonable request.
