# Supplementary material for: Inference of a causal relation between low-density lipoprotein cholesterol and hypertension using mendelian randomization analysis
Source: Clin Hypertens. 2021 Feb 26;27:7. doi: 10.1186/s40885-021-00162-6 (PMC7913402; doi:10.1186/s40885-021-00162-6)
Supplement: Supplementary file 1 — Additional file 1: Supplement Table 1. Associations between genetic polymorphism and LDL-C. [file 40885_2021_162_MOESM1_ESM.docx]

**Supplement**

Supplement table 1. Associations between genetic polymorphism and LDL-C

| SNPs | Genotype |  | LDL-C |  |
| --- | --- | --- | --- | --- |
|  |  | <100 mg/dL | 100-129 mg/dL | ≥130 mg/dL |
| rs41279716 | AA | 26 (32.5) | 34 (42.5) | 20 (25.0) |
|  | AT | 575 (24.9) | 878 (38.0) | 859 (37.2) |
|  | TT | 3621 (21.1) | 6203 (37.2) | 7320 (42.7) |
| rs4970834 | CC | 12 (31.6) | 16 (42.1) | 10 (26.3) |
|  | CT | 432 (27.4) | 624 (39.5) | 523 (33.1) |
|  | TT | 3778 (21.1) | 6475 (36.1) | 7666 (42.8) |
| rs79868705 | GG | 0 (0.0) | 4 (66.7) | 2 (33.3) |
|  | GA | 195 (27.1) | 262 (36.4) | 262 (36.4) |
|  | AA | 4027 (21.4) | 6849 (36.4) | 7935 (42.2) |
| rs79482788 | GG | 0 (0.0) | 4 (66.7) | 2 (33.3) |
|  | GA | 195 (27.1) | 263 (36.5) | 263 (36.5) |
|  | AA | 4027 (21.4) | 6848 (36.4) | 7934 (42.2) |
| rs12740374 | GG | 26 (36.6) | 30 (42.3) | 15 (21.1) |
|  | GT | 594 (26.7) | 865 (38.8) | 770 (34.5) |
|  | TT | 3602 (20.9) | 6220 (36.1) | 7414 (43.0) |
| rs35358959 | GG | 6 (31.6) | 9 (47.4) | 4 (21.1) |
|  | GA | 352 (26.7) | 508 (38.5) | 459 (34.8) |
|  | AA | 3864 (21.2) | 6598 (36.3) | 7736 (42.5) |
| rs672569 | AA | 8 (38.1) | 9 (42.9) | 4 (19.1) |
|  | AG | 358 (26.8) | 513 (38.4) | 465 (34.8) |
|  | GG | 3856 (21.2) | 6593 (36.3) | 7730 (42.5) |
| rs11596737 | GG | 3869 (21.2) | 6620 (36.3) | 7750 (42.5) |
|  | GA | 345 (27.0) | 486 (38.1) | 446 (34.9) |
|  | AA | 8 (40.0) | 9 (45.0) | 3 (15.0) |
| rs17645031 | CC | 5 (33.3) | 9 (60.0) | 1 (6.7) |
|  | CT | 322 (27.1) | 448 (37.7) | 419 (35.2) |
|  | TT | 3895 (21.3) | 6658 (36.3) | 7779 (42.4) |
| rs41306199 | CC | 5 (33.3) | 9 (60.0) | 1 (6.7) |
|  | CT | 322 (27.1) | 448 (37.7) | 419 (35.2) |
|  | TT | 3895 (21.3) | 6658 (36.3) | 7779 (42.4) |
| rs651821 | CC | 1970 (20.2) | 3634 (37.3) | 4144 (42.5) |
|  | CT | 1839 (22.6) | 2899 (35.6) | 3402 (41.8) |
|  | TT | 413 (25.1) | 582 (35.3) | 653 (39.6) |
| rs7952602 | GG | 2424 (22.8) | 3868 (36.3) | 4360 (40.9) |
|  | GC | 1564 (20.7) | 2750 (36.4) | 3251 (43.0) |
|  | CC | 234 (17.7) | 497 (37.7) | 588 (44.6) |
| rs8062041 | CC | 2202 (22.5) | 3689 (37.7) | 3908 (39.9) |
|  | CT | 1636 (20.3) | 2886 (35.9) | 3525 (43.8) |
|  | TT | 384 (22.7) | 540 (32.0) | 766 (45.3) |
| rs2738452 | AA | 3208 (22.2) | 5312 (36.8) | 5908 (41.0) |
|  | AG | 941 (19.9) | 1677 (35.4) | 2117 (44.7) |
|  | GG | 73 (19.6) | 126 (33.8) | 174 (46.7) |
| rs2738464 | GG | 1921 (19.4) | 3493 (35.3) | 4479 (45.3) |
|  | GC | 1850 (23.2) | 2958 (37.2) | 3152 (39.6) |
|  | CC | 451 (26.8) | 664 (39.5) | 568 (33.8) |
| rs892114 | AA | 1144 (22.2) | 1977 (38.3) | 2042 (39.6) |
|  | AG | 2179 (22.1) | 3532 (35.8) | 4155 (42.1) |
|  | GG | 899 (20.0) | 1606 (35.6) | 2002 (44.4) |
| rs6511727 | TT | 1916 (45.4) | 3160 (44.4) | 3437 (41.9) |
|  | GT | 1846 (43.7) | 3161 (44.4) | 3693 (45.0) |
|  | GG | 460 (10.9) | 794 (11.2) | 1069 (13.0) |
| rs387976 | AA | 2171 (19.7) | 4013 (36.3) | 4864 (44.0) |
|  | AC | 1753 (23.9) | 2679 (36.5) | 2913 (39.7) |
|  | CC | 298 (26.1) | 423 (37.0) | 422 (36.9) |
| rs3852861 | GG | 2452 (20.2) | 4461 (36.7) | 5254 (43.2) |
|  | GT | 1535 (23.6) | 2345 (36.0) | 2628 (40.4) |
|  | TT | 235 (27.3) | 309 (35.9) | 317 (36.8) |
| rs7254892 | GG | 31 (62.0) | 13 (26.0) | 6 (12.0) |
|  | GA | 768 (37.4) | 800 (39.0) | 486 (23.7) |
|  | AA | 3423 (19.6) | 6302 (36.2) | 7707 (44.2) |
| rs7412 | CC | 41 (61.2) | 15 (22.4) | 11 (16.4) |
|  | CT | 874 (37.7) | 916 (39.6) | 526 (22.7) |
|  | TT | 3307 (19.3) | 6184 (36.1) | 7662 (44.7) |
| rs445925 | GG | 57 (47.5) | 37 (30.8) | 26 (21.7) |
|  | GA | 950 (33.4) | 1099 (38.6) | 799 (28.1) |
|  | AA | 3215 (19.4) | 5979 (36.1) | 7374 (44.5) |
| rs56131196 | GG | 3526 (22.5) | 5753 (36.6) | 6426 (40.9) |
|  | GA | 650 (18.0) | 1291 (35.8) | 1667 (46.2) |
|  | AA | 46 (20.6) | 71 (31.8) | 106 (47.5) |
| rs7259004 | GG | 218 (28.0) | 276 (35.4) | 285 (36.6) |
|  | GC | 1494 (23.7) | 2335 (37.1) | 2471 (39.2) |
|  | CC | 2510 (20.2) | 4504 (36.2) | 5443 (43.7) |

Values are presented as a number (percentage).

*LDL-C* low-density lipoprotein cholesterol, *SNP* single-nucleotide polymorphism.
